# Origin–entry geometry and left-sided V1 vertebral artery tortuosity: a retrospective cross-sectional CT angiography study

**DOI:** 10.1007/s00234-026-04180-9

**Published:** 2026-09-03

**Authors:** Răzvan Costin Tudose, Rodica Narcisa Calotă, Mugurel Constantin Rusu

**Affiliations:** 1 Division of Anatomy, Carol Davila University of Medicine and Pharmacy, Bucharest, Romania; 2 Research Department, Dr. Carol Davila Central Military Emergency Hospital, Bucharest, Romania

**Keywords:** Vertebral artery, Computed tomography angiography, Anatomic variation, Cervical vertebrae, Cerebral angiography.

## Abstract

**Purpose:**

V1 vertebral artery (VA) tortuosity is associated with cervical artery dissection, stent fracture, and endovascular access difficulty, and predominates on the left; its anatomical basis and its relationship to VA dominance have not been quantitatively tested on CT angiography (CTA).

**Methods:**

Bilateral V1 morphometric analysis was performed on 50 cervical CTAs (24 men, 26 women; 63.2 ± 15.5 years). The tortuosity index (TI) was computed as the centreline-to-straight-line length ratio; VA dominance was determined at two segmental levels.

**Results:**

Left V1 was more tortuous than right (TI 1.37 ± 0.26 vs. 1.20 ± 0.15; Wilcoxon *p* < 0.0001; d_z = 0.74) in 82% of patients. TI correlated with age (Spearman ρ = 0.599, *p* < 0.001 for left; ρ = 0.439, *p* = 0.001 for right) but not with sex, and showed no association with calibre-based dominance (*p* = 0.53). A more cranial VA origin was associated with higher TI (Spearman ρ = −0.64, *p* < 0.001 for left; ρ = −0.37, *p* = 0.009 for right) and remained a significant predictor in multivariable linear regression adjusted for age and sex (left V1 β = −0.0408 per ordinal step toward a more caudal origin, 95% CI − 0.0683 to − 0.0133, *p* = 0.005) against a conserved bilateral C6 transverse-foraminal entry (76%). We refer to this configuration as the origin–entry mismatch.

**Conclusion:**

Left-sided V1 tortuosity was associated with an origin–entry geometric mismatch; no association with calibre-defined dominance was detected.

**Supplementary Information:**

The online version contains supplementary material available at https://doi.org/10.1007/s00234-026-04180-9.

## Introduction

The vertebral artery (VA) is among the most anatomically variable arteries of the human body, and its first segment (V1), extending from the subclavian origin to the bony entry into the cervical transverse foramen, concentrates more of that variability than any other portion of its course [4, 18, 24]. V1 is the segment most exposed during anterolateral cervical approaches and most directly conditions catheter trajectory, stenting outcome and dissection risk [5, 29, 31].

Two parameters dominate descriptive accounts of V1 anatomy: vessel calibre (which underlies the concept of VA dominance) and vessel course (which underlies the concepts of tortuosity, kinking, and coiling). Both have been linked to clinical end-points: dominance chiefly to posterior-circulation ischaemia [14, 23], tortuosity to cervical artery dissection and to endovascular access failure [8, 9, 29, 33], but the two have almost always been treated in isolation. VA dominance is a whole-vessel property, assessed clinically along the entire course of the vessel and by its contribution to the basilar artery, rather than within a single segment. Whether segment-level V1 geometry nonetheless tracks that whole-vessel property, and whether either is better understood as the consequence of a third, more fundamental anatomical variable, has not been formally tested with bilateral, paired measurements.

Prior anatomical work has documented variability in V1 origin level (subclavian artery in the great majority, with anomalous aortic-arch origin in 4–13% of cases) [3, 7, 24, 30], variability in the level of transverse foraminal entry (most often C6, less commonly C5 or C7) [24, 28, 30], and a pooled left-sided dominance prevalence of approximately one in three [16, 20, 24]. Tortuosity has been quantified separately, primarily in the carotid system [8, 21, 25, 27, 32], with the centreline-to-straight-line ratio (the tortuosity index, TI) emerging as the most reproducible quantitative measure. To our knowledge, no study has integrated these strands, bilateral V1 morphometry, granular origin and entry level, and tortuosity quantification, into a single dataset on the same patients.

The present study addresses these gaps. We measured bilateral V1 tortuosity, calibre, origin level, and transverse foraminal entry level in 50 cervical CT angiograms with anatomically normal VAs and tested the relationships among these variables in a pre-specified analysis. We hypothesised, in advance, (i) that V1 tortuosity would show left-sided predominance, (ii) that this predominance would be better explained by an origin–entry geometric mismatch than by vessel calibre dominance. The results support both hypotheses and motivate a structurally informed re-conceptualisation of V1 tortuosity.

## Materials and methods

### Study design and ethical approval

This was a single-centre, retrospective, observational anatomical-imaging study. The research was conducted ethically in accordance with the World Medical Association Declaration of Helsinki (the Code of Ethics). The responsible institutional ethics committee approved the study. Written informed consent was obtained from every patient prior to the diagnostic CT examination, including consent for anonymised retrospective use of their imaging data for research purposes. The reporting follows the recommendations of the Strengthening the Reporting of Observational Studies in Epidemiology (STROBE) statement for observational studies [26].

### Patient selection

Archived cervico-cranial CT angiography (CTA) examinations were retrieved from the institutional Picture Archiving and Communication System (PACS). Studies had been performed for the diagnostic evaluation of carotid arterial pathology and were re-purposed for the present anatomical investigation. Inclusion criteria were: (i) bilateral V1 segments fully opacified and visible from the origin (subclavian artery) to the cervical transverse foramen entry on the source dataset; (ii) absence of VA pathology on the index examination, including dissection, occlusion, ≥ 50% stenosis, hypoplasia (lumen diameter < 2 mm), thrombus, or arteriovenous malformation; (iii) absence of prior cervical or vascular surgery affecting the supra-aortic course of the VA; and (iv) absence of any documented connective-tissue disorder. Patients with carotid pathology, the original clinical indication, were not excluded, since the disease was confined to the carotid system and did not involve the VA territory.

**Fig. 1:**
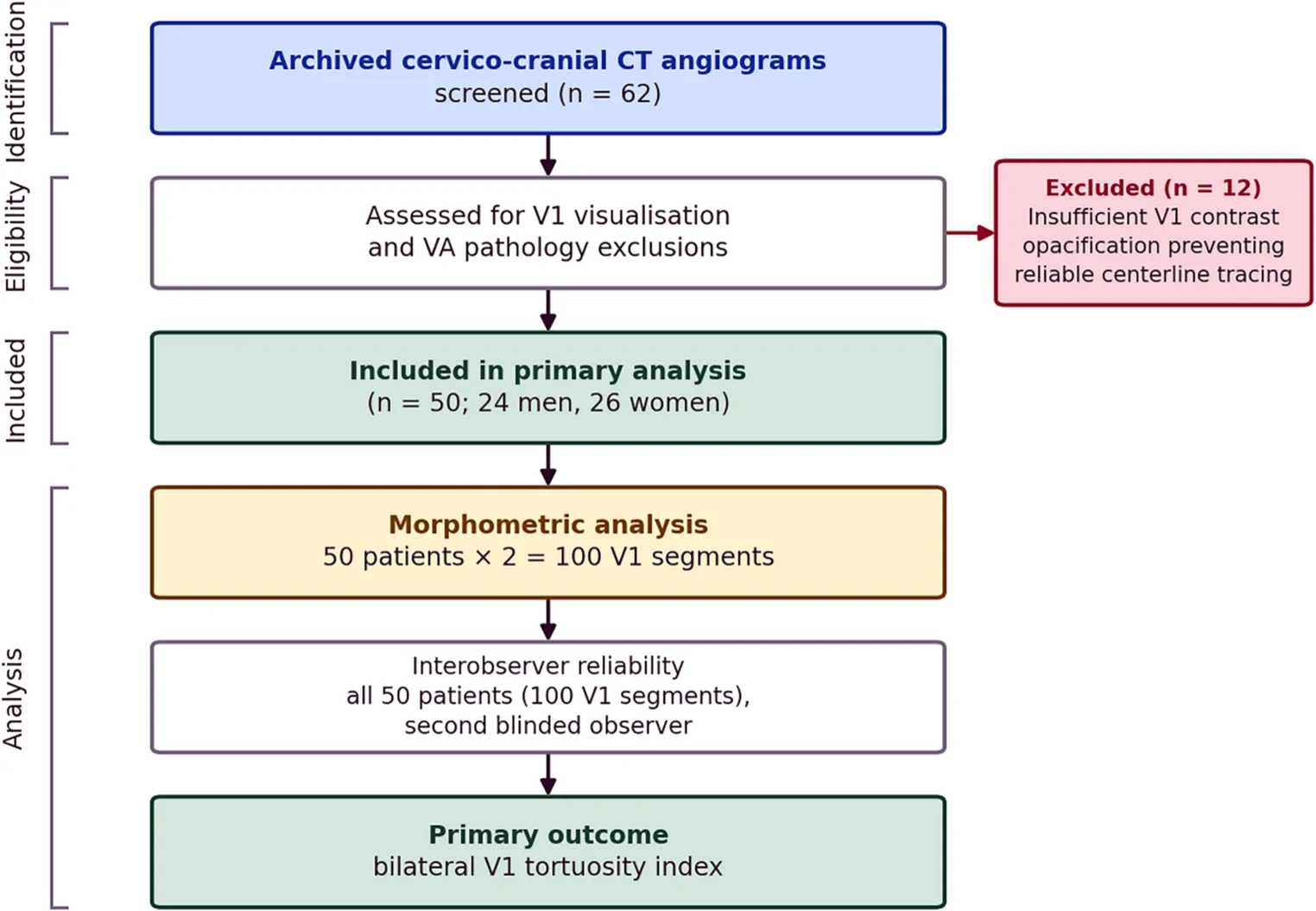
Flow diagram of patient identification, eligibility assessment, and inclusion

A total of 62 archived examinations were screened; 12 were excluded because of insufficient V1 contrast opacification that prevented reliable centreline tracing. The final analytic cohort comprised 50 patients (24 men, 26 women; mean age 63.2 ± 15.5 years; range 34–90). The flow of cases through the study is summarised in Fig. 1.

### CT angiography acquisition and image reconstruction

All examinations were performed on a single 32-slice multi-detector CT scanner (Siemens SOMATOM Perspective, Siemens Healthineers, Forchheim, Germany) using the institutional standardised cervico-cranial CTA protocol. Acquisition and reconstruction parameters are reported in full in Online Resource 1 (Table S1) in line with current best practice for cervical CTA in the evaluation of cerebrovascular pathology [6]. Source data were reconstructed at 0.75 mm with 50% inter-slice overlap to yield near-isotropic voxels suitable for multi-planar reformation and three-dimensional volume rendering.

### Image post-processing and morphometric measurements

#### Software environment

All post-processing and measurements were performed in Horos (version 4.0; The Horos Project, https://horosproject.org), an open-source DICOM viewer derived from OsiriX. Horos provides multi-planar reformation, MIP, 3D volume rendering and curved-MPR, and its measurement tools have been validated against vendor workstations [12]. Measurements were made on a calibrated diagnostic monitor under standardised reading conditions; full display and post-processing settings are given in Online Resource 1 (Supplementary Methods).

#### Manual centreline tracing of the V1 vertebral artery segment

**Fig. 2:**
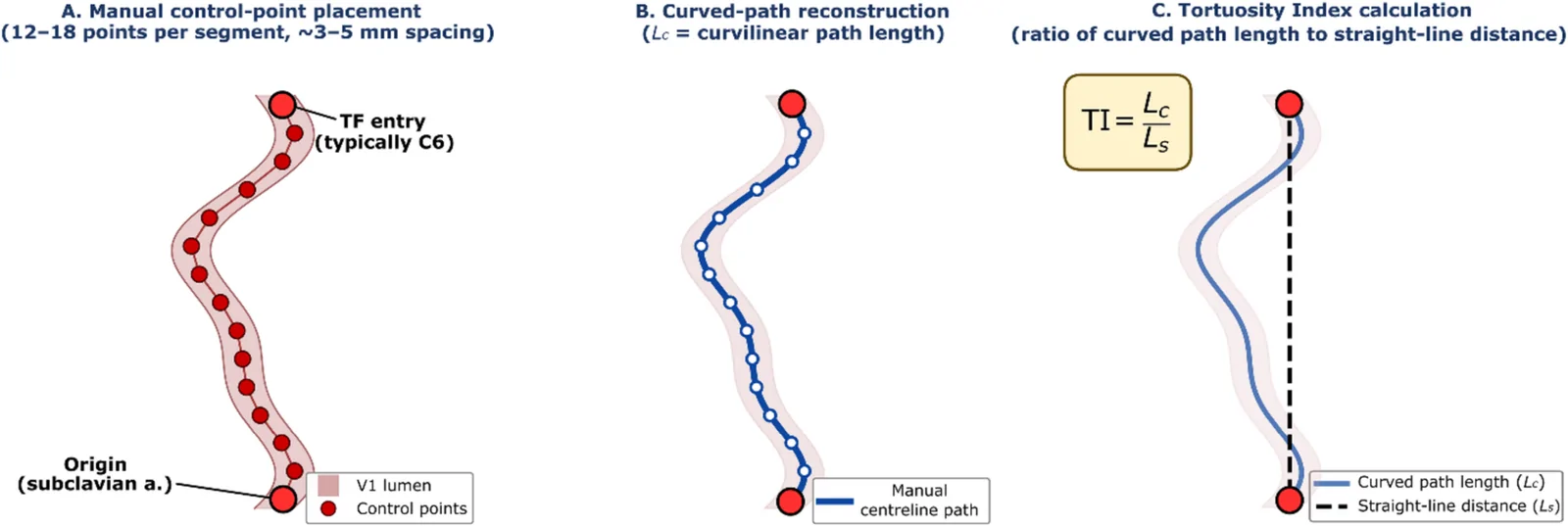
Schematic representation of the V1 vertebral artery tortuosity measurement protocol. (**A**) Sequential control points (12–18 per V1 segment) were manually placed along the centre of the contrast-enhanced V1 lumen from the vertebral artery origin to the transverse foramen entry point, with denser spacing in regions of curvature. (**B**) A manual curved path was reconstructed through the control points using the Horos curved-MPR/path tool and used as the operational centreline for curvilinear length measurement (L_c_). (**C**) The tortuosity index was calculated as the ratio between the curved path length (L_c_) and the direct endpoint-to-endpoint straight-line distance (L_s_)

The V1 segment was defined, in accordance with established anatomical convention [31], as the portion of the VA extending from its origin at the subclavian artery to its entry into the transverse foramen of the corresponding cervical vertebra. The centreline of each V1 segment was traced manually by a single observer using a standardised, multi-step protocol designed to maximise spatial accuracy and reproducibility (Fig. 2).

In brief, the proximal endpoint was placed at the centre of the VA ostium and the distal endpoint at the centre of the lumen on the slice at which the vessel first entered the transverse foramen; 12–18 control points were placed at 3–5 mm intervals (2 mm through pronounced curvature) at the geometric centre of the lumen on cross-sections perpendicular to the local vessel axis; the resulting curved path was inspected and adjusted in three planes and used as the operational centreline. Centreline length (Lc) was the curvilinear length of that path and straight-line length (Ls) the direct endpoint-to-endpoint distance on a standardised oblique multiplanar reconstruction. The tortuosity index was TI = Lc/Ls. The full step-by-step protocol, which follows established methodology for cervical vascular morphometry [8, 21, 25, 27, 32] is provided in Online Resource 1 (Supplementary Methods).

#### Vertebral artery diameter measurement and dominance classification

Bilateral lumen diameters were measured at two pre-specified anatomical levels: (i) the V1–V2 transition, defined as the cross-section immediately distal to the entry of the VA into the transverse foramen (typically C6); and (ii) the mid-V2 segment, taken at the level of the C4 transverse foramen. At each level, an axial cross-section perpendicular to the local long axis of the artery was first reconstructed in Horos, and lumen diameters were measured along two orthogonal axes; the mean of the two measurements was retained as the lumen diameter at that level. Dominance was classified at each of the two levels independently using the pre-specified threshold of ≥ 0.3 mm absolute bilateral diameter difference, in accordance with widely accepted neuroradiological convention [16, 20, 24]. Patients with a bilateral diameter difference < 0.3 mm at both levels were classified as codominant. The agreement between dominance classifications at the two levels was reported as a separate outcome (see Sect.  2.6).

#### Tortuosity index calculation

**Fig. 3:**
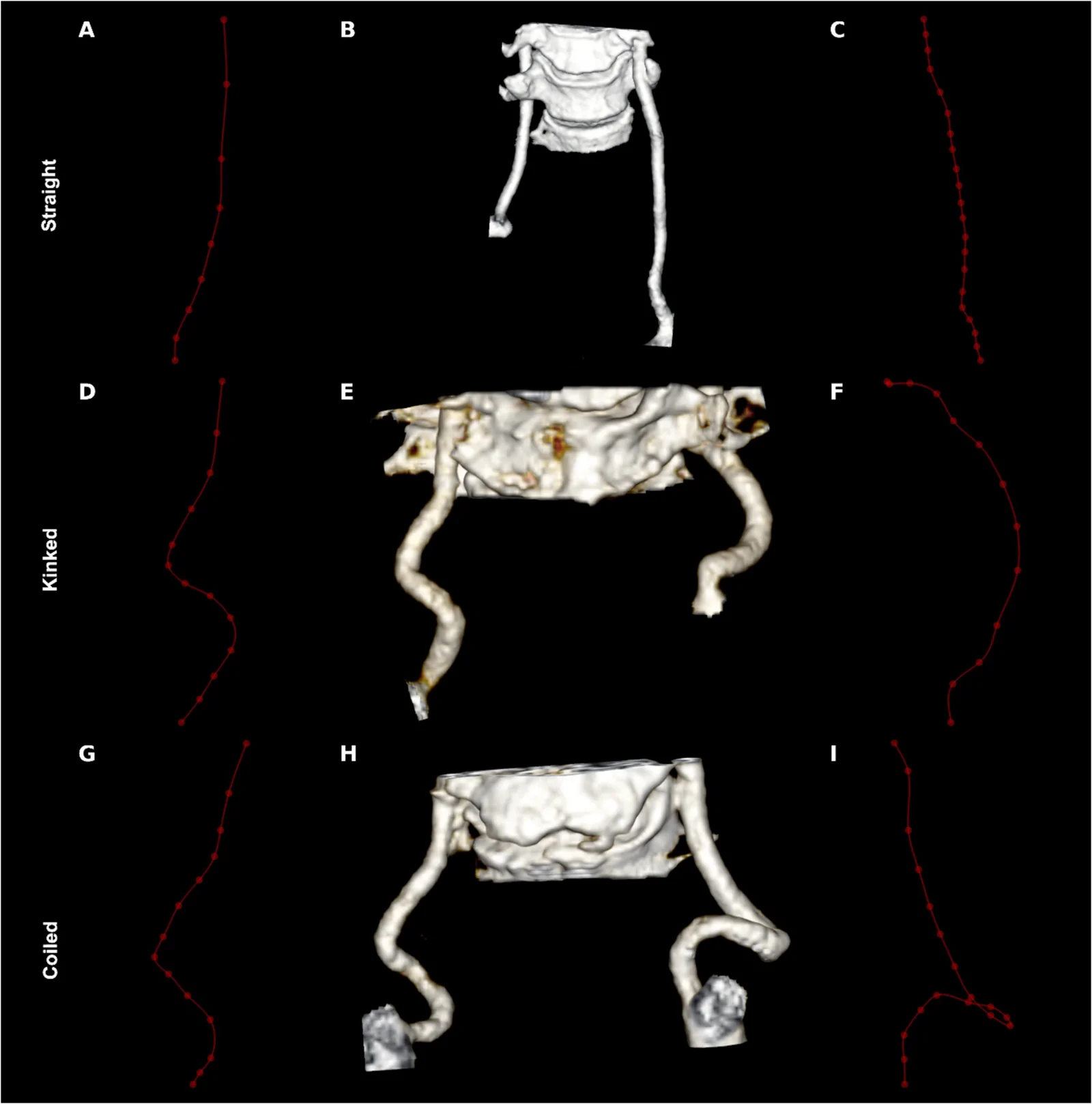
Visual reference for the three V1 vertebral artery tortuosity categories used throughout the study. Rows correspond to the three pre-defined categories: *straight* (TI < 1.10; panels **A**–**C**), *kinked* (TI 1.10–1.30; panels **D**–**F**), and *coiled* (TI ≥ 1.30; panels **G**–**I**). Each row shows the same patient case as the stand-alone right V1 centreline pathway tracing (left column; **A**, **D**, **G**), the anterior 3D volume-rendered CTA reconstruction of the bilateral V1 segments with the surrounding cervical vertebrae (centre column; **B**, **E**, **H**), and the stand-alone left V1 centreline pathway tracing (right column; **C**, **F**, **I**). Pathway tracings are rendered as smooth red curves passing through the manually placed control points against a uniform black background

The V1 TI was calculated for each side as TI = L_c_/L_s_, where L_c_ and L_s_ are the centreline and straight-line lengths derived as described in Sect.  2.4.2. Geometrically, TI ≥ 1.00 by construction, with TI = 1.00 corresponding to a perfectly straight vessel. TI categories used for descriptive reporting followed the conventional classification of cervical vascular tortuosity [27, 32]: straight (TI < 1.10), kinked (TI 1.10–1.30), and coiled (TI ≥ 1.30). Representative bilateral examples of each category are shown in Fig. 3, with the anterior 3D volume-rendered CTA reconstruction and the stand-alone right and left V1 centreline pathway tracings displayed side-by-side for direct visual reference.

#### Vertebral artery origin level

The vertebral level of origin of the VA from the subclavian artery was determined separately for each side using a granular ordinal scale that combined the vertebral body and intravertebral sub-level. The vertebral body adjacent to the VA ostium was identified on sagittal cross-sections (C6, C7, T1, T2, or T3). In each case, the origin level was assigned by projecting the VA ostium orthogonally to the adjacent vertebral column, ensuring correct level attribution even in patients with accentuated cervical or cervicothoracic curvature. Within each vertebral body, the sub-level was further specified as the superior, middle, or inferior third of body height. Origins lying directly at the intervertebral disc level (e.g., C7–T1, T1–T2) were coded as junctional origins. This granular classification was then mapped to a continuous ordinal index, with smaller values denoting more cranial origins and larger values denoting more caudal origins, for use in subsequent correlation and regression analyses.

#### Transverse foramen entry level

The level at which each VA entered the bony transverse foraminal canal was determined separately for both sides, on axial cross-sections, by identifying the most caudal cross-section in which the artery was fully encircled by the bony rim of the transverse foramen. Entry was coded as one of C4, C5, C6, or C7. Bilateral concordance and the proportion of variant entry levels were reported as descriptive outcomes.

### Interobserver reliability

Interobserver reliability of the morphometric measurements was assessed in the complete cohort (*n* = 50 patients; 100 V1 segments). The second observer, a senior anatomist with more than 20 years of expertise in vascular and head-and-neck anatomical research, independently re-traced the bilateral V1 centrelines using the same protocol described in Sect.  2.4.2. The second observer was blinded to the first observer’s control-point positions, reconstructed path, and numerical measurements. The interval between the first and second observer’s tracings was at least two weeks. Centreline length, straight-line length, and tortuosity index were re-derived independently from the second observer’s tracings, and bilateral measurements from the two observers were used to compute interobserver reliability statistics as detailed in Sect.  2.6. Centreline length, straight-line length and the derived tortuosity index were re-derived independently for both sides in every patient from the second observer’s tracings; these three variables were the parameters compared between the two raters.

### Statistical analysis

#### Software

All statistical analyses were performed in Python (version 3.12; Python Software Foundation) using the SciPy stack, specifically SciPy (version ≥ 1.13), statsmodels (version ≥ 0.14), pingouin (version ≥ 0.5.4), and scikit-learn (version ≥ 1.4). Plotting was performed in Matplotlib (version ≥ 3.8). All analysis scripts are available from the corresponding author on reasonable request.

#### Descriptive statistics and distributional checks

Continuous variables are reported as mean ± standard deviation, supplemented by the median and interquartile range (IQR) for variables that were not normally distributed. Categorical variables are reported as counts and percentages. Distributional normality was tested with the Shapiro–Wilk test for each continuous variable; non-parametric methods were preferred whenever the Shapiro–Wilk test rejected the normality null at α = 0.05, and parametric methods were used as confirmatory comparators where applicable.

#### Bilateral paired comparisons

Within-patient bilateral comparisons (right vs. left V1 measurements) were conducted using the Wilcoxon signed-rank test as the primary comparator; the paired-samples t-test was reported as a parametric confirmatory test. Within-pair effect size was quantified using Cohen’s d_z_. The directional consistency of each bilateral comparison was additionally assessed using the exact sign test on the proportion of patients in whom the left-side measurement exceeded the right-side measurement. To ensure that the primary bilateral finding was not contingent on distributional assumptions, the bilateral mean difference and Cohen’s d_z_ were also re-estimated using a non-parametric percentile bootstrap (10,000 resamples drawn with replacement from the paired-difference distribution); the corresponding 95% confidence intervals were reported as a sensitivity analysis.

#### Correlations and multivariable regression

Bivariate associations between continuous variables (e.g., age and tortuosity index) were quantified using Spearman’s rank correlation coefficient (ρ), with the corresponding asymptotic two-sided p-value reported. Bivariate associations between categorical (or ordinal) and continuous variables were assessed with the Mann–Whitney U test (two-group comparisons) or the Kruskal–Wallis H test (three-or-more-group comparisons).

Multivariable linear regression was used to assess the joint and independent contributions of pre-specified predictors to the V1 TI. Two models were fitted, one per side: the dependent variable was the side-specific V1 TI; the independent variables were age (continuous, in years), sex (male = 1, female = 0), and origin level (granular ordinal index as defined in Sect.  2.4.5; positive values denoting more caudal origins). Multicollinearity among the predictors was assessed by computing the variance inflation factor (VIF) for each variable in each model after inclusion of the regression intercept; all VIFs were below 1.25, well within the conventional threshold of 5 (and far below the threshold of 10), confirming the absence of meaningful multicollinearity. Residual normality and homoscedasticity were assessed using normal probability plots and residual-versus-fitted plots; no violations were identified that would have warranted transformation of the dependent variable. For each predictor, the unstandardised regression coefficient (β), its standard error, the 95% confidence interval, and the asymptotic p-value are reported.

#### Interobserver reliability statistics

Interobserver reliability was quantified using the intraclass correlation coefficient with two-way mixed-effects, single-rater, absolute-agreement specification. The 95% confidence interval for each ICC was estimated by the F-distribution method and reported alongside the point estimate. In addition to the ICC, Bland–Altman analysis was performed for the primary outcome (V1 TI) to characterise the magnitude and direction of inter-observer disagreement at the level of individual measurements; bias and 95% limits of agreement (LoA = bias ± 1.96 × SD of differences) are reported separately for the right and the left V1 [2]. ICC(A,1) [17, 22] was computed separately for the right and left V1 centreline length, straight-line length and tortuosity index, and interpreted using established thresholds [15]. VA dominance, origin level and transverse foraminal entry level were assigned by the primary observer only and were not subject to interobserver comparison. Agreement between the VA dominance classifications made at the two segmental levels was assessed separately, in the whole cohort, using Cohen’s unweighted κ.

#### Significance threshold, power justification, and pre-specification

All statistical tests were two-sided unless otherwise stated, and the conventional significance threshold of α = 0.05 was applied throughout. No multiplicity correction was applied across primary outcomes for the morphometric arm, because each primary outcome (bilateral TI asymmetry, age-related TI progression, origin-level association with TI, and dominance independence of TI) was a pre-specified separate research question rather than a member of a single test family.

With a sample of 50 paired observations and α = 0.05 (two-sided), the study was powered at approximately 0.79 to detect within-pair effects of Cohen’s d_z_ = 0.40 (a small-to-medium effect by conventional benchmarks) in paired comparisons; power exceeded 0.93 for d_z_ = 0.50 and reached 1.00 for d_z_ = 0.74 (the actually observed primary effect). The sample size was therefore well matched for detecting clinically and anatomically meaningful bilateral asymmetries, while remaining conservative in detecting smaller secondary effects.

The primary research questions, the primary statistical tests, the inclusion and exclusion criteria, the dominance threshold, and the tortuosity-classification cut-points were all specified in writing prior to the start of any analysis. The dataset was locked before unblinding the second observer’s measurements.

## Results

### Patient sample

The final cohort comprised 50 patients (24 men, 26 women) with a mean age of 63.2 ± 15.5 years (median 61.5; range 34–90). Of 62 examinations screened, 12 were excluded due to insufficient V1 contrast opacification preventing reliable centreline tracing; no further exclusions were necessary on the basis of connective-tissue disorder, prior cervical vascular surgery, or pre-existing VA pathology. Age did not differ between sexes (Mann–Whitney U = 278.5, *p* = 0.521). Demographic and dominance characteristics are summarised in Table 1.

**Table 1:** Patient demographics, vertebral artery dominance distribution, and two-level dominance consistency. SD = standard deviation; IQR = interquartile range. Dominance was defined as a bilateral vertebral artery diameter difference of ≥ 0.3 mm; patients below that threshold at both sampled levels were classified as codominant. Two-level dominance concordance denotes agreement between the classification made at the V1–V2 transition and that made at mid-V2 (C4)

Variable	Total (*n* = 50)	Men (*n* = 24)	Women (*n* = 26)
Age, mean ± SD (years)	63.2 ± 15.5	62.8 ± 14.7	63.6 ± 16.4
Age, median [IQR] (years)	61.5 [52.0–76.0]	62.0 [51.5–74.0]	61.5 [54.0–78.5]
Right-dominant, n (%)	25 (50.0)	13 (54.2)	12 (46.2)
Left-dominant, n (%)	16 (32.0)	8 (33.3)	8 (30.8)
Codominant, n (%)	9 (18.0)	3 (12.5)	6 (23.0)
Two-level dominance concordance, n (%)	50 (100.0)	24 (100.0)	26 (100.0)

### Interobserver reliability

Interobserver reliability was assessed in the complete cohort (*n* = 50 patients, 100 V1 segments), each segment being re-measured independently by a second blinded observer. Agreement on the primary outcome, bilateral V1 tortuosity index (TI), was excellent: ICC(A,1) = 0.980 (95% CI 0.965–0.989; *p* < 0.001) for the right V1 and ICC(A,1) = 0.986 (95% CI 0.976–0.992; *p* < 0.001) for the left V1. Bland–Altman analysis showed negligible systematic bias (right V1: bias − 0.005, 95% limits of agreement [LoA] − 0.064 to + 0.055; left V1: bias − 0.005, 95% LoA − 0.093 to + 0.083). Agreement on the underlying length measurements was likewise excellent: centreline length ICC(A,1) = 0.998 (95% CI 0.996–0.999) right and 0.973 (95% CI 0.953–0.985) left; straight-line length ICC(A,1) = 0.997 (95% CI 0.995–0.999) right and 0.987 (95% CI 0.977–0.992) left. VA dominance, origin level and transverse foraminal entry level were assigned by the primary observer only.

### Vertebral artery origin and transverse foramen entry

The VA origin level was strongly asymmetric. The right VA originated at the level of the T1 vertebral body in 38/50 patients (76.0%), at C7 in 5/50 (10.0%), at T2 in 6/50 (12.0%), and at C6 in 1/50 (2.0%). The left VA, in contrast, showed a wider distribution: T1 in 29/50 (58.0%), T2 in 10/50 (20.0%), C7 (including C7–T1 junctions) in 8/50 (16.0%), and T3 in 3/50 (6.0%). The origin level of the left VA was discordant with that of the right VA in 25/50 patients (50.0%); in 8 of these, the left VA originated more cranially than the right, whereas in 12, it originated more caudally (5 at the same body level but at a different sub-level).

Transverse foramen entry, by contrast, was substantially more conserved. Bilateral C6 entry was present in 38/50 patients (76.0%); an additional 3 patients (6.0%) had bilateral C5 or C7 entry. Asymmetric entry occurred in 9 patients (18.0%), with variant entry levels concentrated on the right side (10 vs. 5 variant sides among 100 evaluated VAs). No combination of variant entry levels showed any deviation from the expected anatomy of the transverse foramen.

### Vertebral artery diameter and dominance

Bilateral diameter measurements showed no significant side-to-side difference at either level: right V1–V2 diameter 3.61 ± 0.76 mm vs. left V1–V2 diameter 3.49 ± 0.73 mm (Wilcoxon *p* = 0.275); right mid-V2 diameter 3.50 ± 0.76 mm vs. left mid-V2 diameter 3.43 ± 0.72 mm (Wilcoxon *p* = 0.310). The mean side-to-side absolute diameter difference at the V1–V2 transition was 0.62 ± 0.62 mm (range 0.01–3.32 mm), and the maximum-to-minimum diameter ratio was 1.32 ± 0.39 (range 1.01–3.23).

Applying the pre-specified threshold of ≥ 0.3 mm bilateral diameter asymmetry, 25/50 patients (50.0%) were right-dominant, 16/50 (32.0%) were left-dominant, and 9/50 (18.0%) were codominant. VA dominance was classified independently at two segmental levels, the V1–V2 transition and mid-V2 (C4). The two classifications were compared with Cohen’s κ and agreed in every patient (50/50; κ = 1.00), with no case of cross-segmental dominance reversal. This supports the stability of the dominance classification across the two sampled levels in the present cohort; it does not, however, establish dominance as a whole-vessel property, which would require assessment along the entire V1–V4 course and of the contribution to the basilar artery.

Bilateral V1 morphometric measurements are summarised in Table 2.

**Table 2:** Bilateral V1 segment morphometric measurements with paired comparisons. p-values from the Wilcoxon signed-rank test. Values are mean ± SD. SD = standard deviation; TI = tortuosity index, calculated as centreline length divided by straight-line length; V1–V2 = transition between the first and second vertebral artery segments; C4 = fourth cervical vertebra. p-values are from the Wilcoxon signed-rank test for paired samples

Measurement	Right V1	Left V1	Wilcoxon *p*
Centreline length (cm)	5.29 ± 1.33	5.61 ± 1.25	0.026
Straight-line length (cm)	4.48 ± 1.23	4.26 ± 1.38	0.074
Tortuosity index (TI)	**1.20 ± 0.15**	**1.37 ± 0.26**	**< 0.0001**
V1–V2 transition diameter (mm)	3.61 ± 0.76	3.49 ± 0.73	0.275
Mid-V2 (C4) diameter (mm)	3.50 ± 0.76	3.43 ± 0.72	0.310

### V1 vertebral artery tortuosity index – bilateral asymmetry

**Fig. 4:**
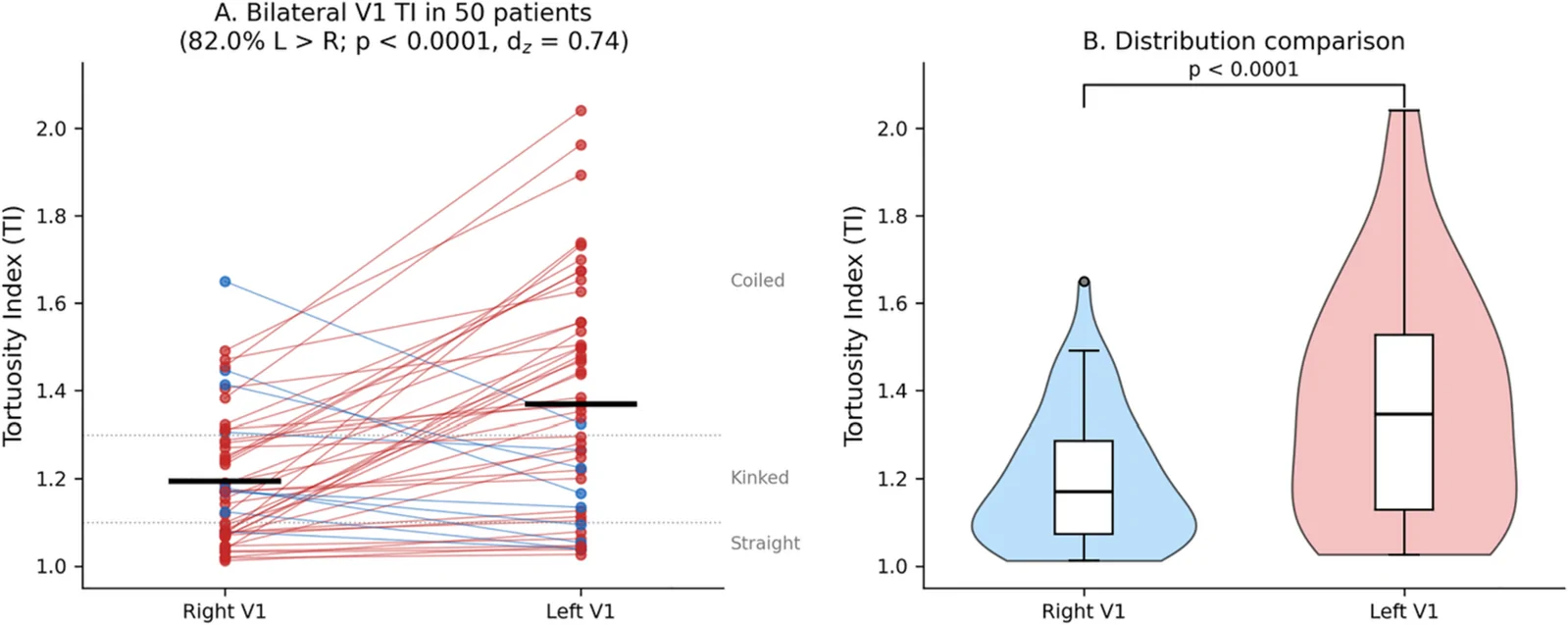
Bilateral V1 tortuosity index distribution across the cohort (*n* = 50). (**A**) Paired plot connecting right and left V1 TI for every patient; red lines indicate L > R, blue lines indicate R > L. Black bars represent group means. Reference dotted lines mark the conventional straight (TI < 1.10), kinked (TI 1.10–1.30), and coiled (TI ≥ 1.30) categories. (**B**) Violin–box plot summary; medians (centre lines), interquartile ranges (boxes), and 1.5 × IQR whiskers are shown. d_z_ = within-pair (paired) Cohen’s effect size

Across the full cohort, the left V1 was substantially more tortuous than the right (Fig. 4). Left V1 TI averaged 1.37 ± 0.26 (median 1.35; IQR 1.13–1.53; range 1.03–2.04), whereas right V1 TI averaged 1.20 ± 0.15 (median 1.17; IQR 1.07–1.29; range 1.01–1.65). Both distributions deviated from normality (Shapiro–Wilk: right *p* < 0.001; left *p* = 0.017). The paired Wilcoxon signed-rank test confirmed a highly significant left-sided predominance (W = 180.0, *p* < 0.0001), corroborated by a paired-samples t-test (t = − 5.24, *p* < 0.0001) and a within-pair effect size (Cohen’s d_z_) of 0.74. The direction of the asymmetry was consistent: the left V1 was more tortuous than the right in 41/50 patients (82.0%), and reversed in 9/50 (18.0%), with no ties; the corresponding sign test yielded *p* = 5.6 × 10⁻⁶. Non-parametric percentile bootstrap (10,000 resamples) gave a mean bilateral difference of 0.174 (95% CI 0.110 to 0.239) and d_z = 0.74 (95% CI 0.48 to 1.06).

At the cohort level, the bilateral asymmetry reflects both a significant excess in centreline length on the left (5.61 ± 1.25 cm vs. right 5.29 ± 1.33 cm; Wilcoxon *p* = 0.026) and a smaller, non-significant reduction in straight-line distance (left 4.26 ± 1.38 cm vs. right 4.48 ± 1.23 cm; *p* = 0.074); both differences act in the direction of a higher left V1 TI. Exact sign tests for the remaining bilateral comparisons were as follows: centreline length, left greater in 35/50 (70.0%), *p* = 0.007; straight-line length, left greater in 20/50 (40.0%), *p* = 0.203; V1–V2 transition diameter, left greater in 20/50 (40.0%), *p* = 0.203; mid-V2 diameter, left greater in 19/49 evaluable pairs (38.8%), *p* = 0.152. All other variables were complete for all 50 patients; no imputation was performed.

### V1 tortuosity index and demographic factors

**Fig. 5:**
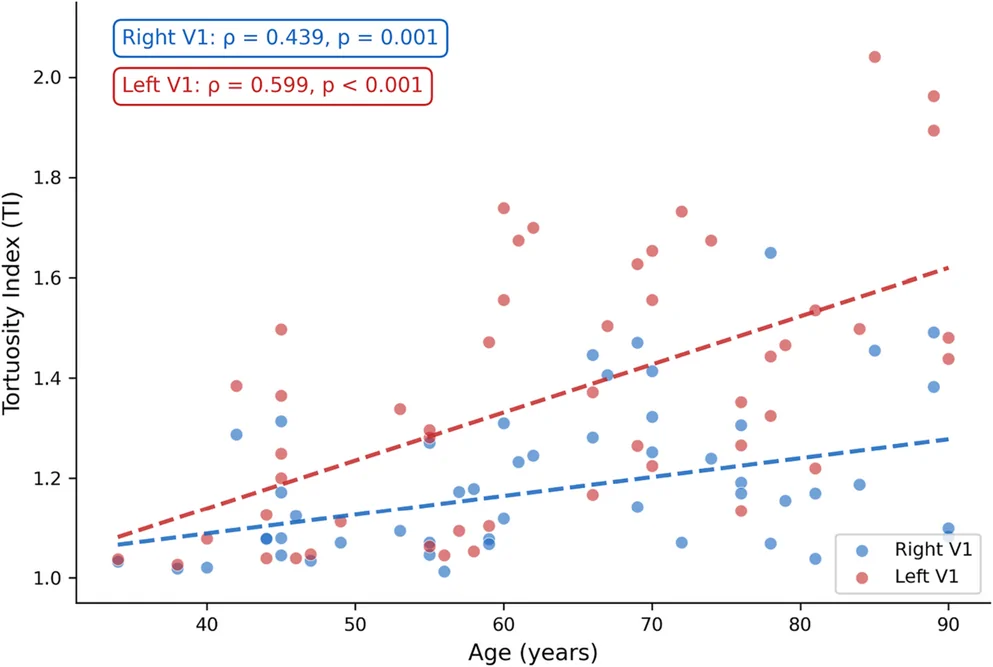
Age-related progression of V1 vertebral artery tortuosity. Each point represents one V1 segment; dashed lines are robust (Theil–Sen) regression fits. Left V1 (red) shows a steeper age-related slope than right V1 (blue), and Spearman correlations are stronger on the left, consistent with the larger absolute tortuosity values found on that side

V1 TI showed a strong, monotonic, age-dependent increase, with greater left-sided predominance. Spearman correlation between age and TI was ρ = 0.439 (*p* = 0.001) for the right V1 and ρ = 0.599 (*p* < 0.001) for the left V1 (Fig. 5). When the cohort was stratified at age 60, a conventional cut-off for studies of acquired vascular remodelling, both sides showed significantly higher TI values in the older subgroup (right V1: 1.11 ± 0.09 in patients < 60 vs. 1.26 ± 0.15 in patients ≥ 60, *p* < 0.001; left V1: 1.18 ± 0.15 vs. 1.52 ± 0.24, *p* < 0.001). The bilateral L > R asymmetry remained statistically significant within each age stratum (under-60 stratum: Wilcoxon *p* = 0.019; 60-and-over stratum: *p* < 0.001). In contrast, sex had no detectable influence on either side. Right V1 TI was 1.18 ± 0.14 in men versus 1.21 ± 0.16 in women (U = 276.0, *p* = 0.491); left V1 TI was 1.36 ± 0.28 in men versus 1.38 ± 0.25 in women (U = 303.0, *p* = 0.869).

### V1 tortuosity index and vertebral artery origin level

**Fig. 6:**
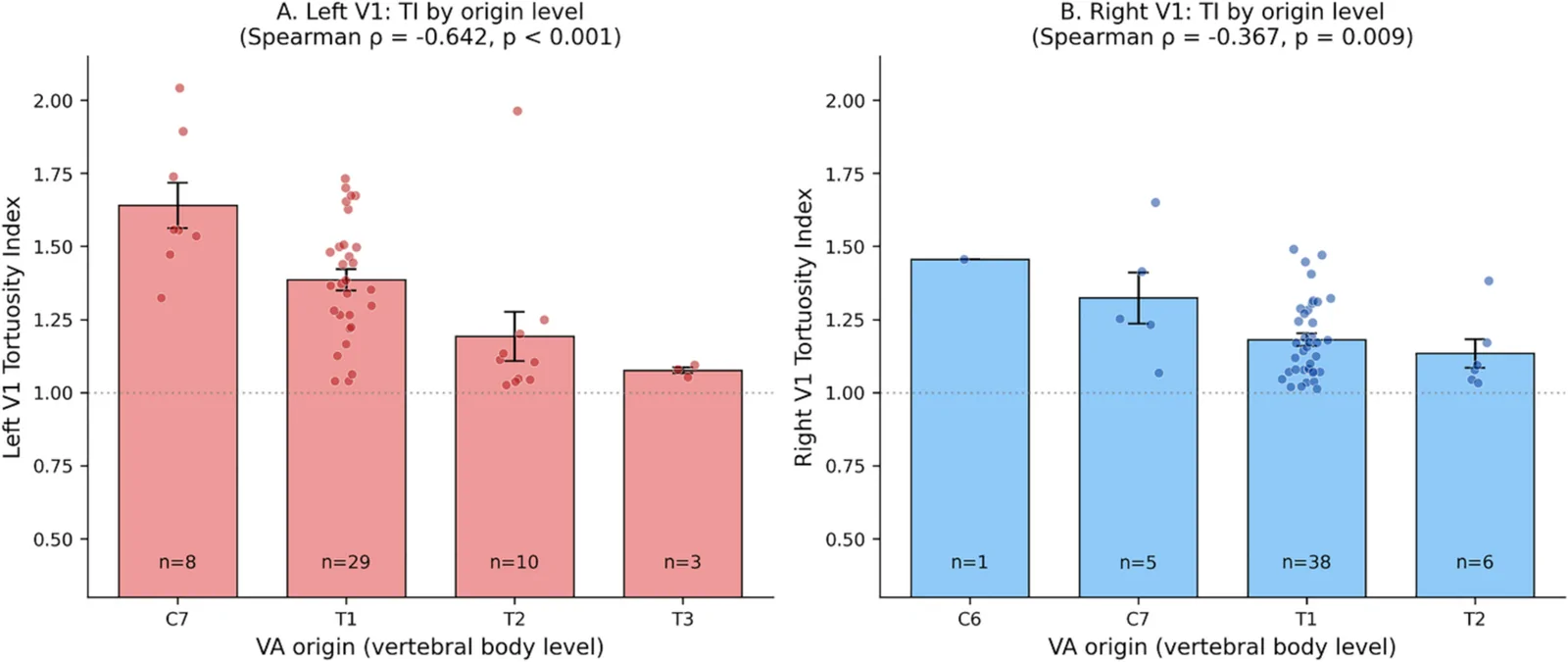
V1 tortuosity index stratified by vertebral artery origin level. (**A**) Left V1: a clear monotonic decrease in TI from C7 to T3 origins (Spearman ρ = −0.642, *p* < 0.001). (**B**) Right V1: the same direction of effect at a lower magnitude (ρ = −0.367, *p* = 0.009). Bars represent mean ± SEM; individual values are overlaid as jittered points. The dotted line at TI = 1.0 indicates a perfectly straight vessel

V1 TI was analysed after stratification of the cohort by VA origin level. On the left side, mean L V1 TI rose progressively as the VA origin moved from caudal to cranial vertebral bodies: T3 origin (*n* = 3) – TI 1.08 ± 0.02; T2 origin (*n* = 10) – TI 1.20 ± 0.30; T1 origin (*n* = 29) – TI 1.39 ± 0.20; C7 origin including C7–T1 junction (*n* = 8) – TI 1.64 ± 0.24. The Spearman correlation between origin level (ranked from cranial to caudal) and left V1 TI was ρ = −0.642 (*p* < 0.001) using granular sub-level positions, and the Kruskal–Wallis test across the four left-side origin groups was H = 18.67, *p* < 0.001. The same gradient was present, although less pronounced, on the right side: T2 origin (*n* = 6) – TI 1.13 ± 0.13; T1 origin (*n* = 38) – TI 1.19 ± 0.14; C7 origin (*n* = 5) – TI 1.32 ± 0.22; the single C6 origin showed TI 1.46 (Spearman ρ = −0.367, *p* = 0.009) (Fig. 6). Bilateral tortuosity indices and mean centreline lengths by origin level are given in Table 3.

**Table 3:** Distribution of V1 tortuosity index by vertebral artery origin level. TI = tortuosity index; SD = standard deviation; n = number of patients; C = cervical vertebra; T = thoracic vertebra. No SD is given for the right C6 group because it contains a single patient

Side	Origin	*n*	Mean TI ± SD	Centreline (cm)
Left V1	C7	8	1.64 ± 0.24	4.88
	T1	29	1.39 ± 0.20	5.38
	T2	10	1.20 ± 0.30	5.83
	T3	3	1.08 ± 0.02	9.04
Right V1	C6	1	1.46	7.86
	C7	5	1.32 ± 0.22	4.74
	T1	38	1.19 ± 0.14	5.24
	T2	6	1.13 ± 0.13	5.63

### Multivariable predictors of V1 tortuosity index

Multivariable linear regression models were fitted separately for left and right V1 TI, with age (continuous), sex (male = 1), and origin level (granular ordinal scale, caudal direction positive) as predictors (Table 4).

**Table 4:** Multivariable linear regression models for left and right V1 tortuosity index. β values are unstandardised regression coefficients. β = unstandardised regression coefficient; SE = standard error; CI = confidence interval; R² = coefficient of determination; adj R² = adjusted coefficient of determination; M = male. Origin level is the granular ordinal index defined in Sect.  2.4.5, with positive values denoting more caudal origins. Left V1 model: F(3, 46) = 13.11, *p* < 0.001. Right V1 model: F(3, 46) = 5.73, *p* = 0.002

Side / Predictor	β	SE	95% CI	*p*
Left V1 (R² = 0.461; adj R² = 0.426)				
Age (per year)	0.0081	0.0020	0.0041 to 0.0121	**< 0.001**
Sex (M = 1)	−0.027	0.061	−0.150 to 0.096	0.655
Origin level (caudal positive)	−0.0408	0.0136	−0.0683 to − 0.0133	**0.005**
Right V1 (R² = 0.272; adj R² = 0.225)				
Age (per year)	0.0039	0.0013	0.0014 to 0.0064	**0.003**
Sex (M = 1)	−0.013	0.039	−0.092 to 0.067	0.749
Origin level (caudal positive)	−0.0346	0.0153	−0.0654 to − 0.0038	**0.029**

For the left V1 TI model, both age (β = 0.0081 per year, 95% CI 0.0041 to 0.0121, *p* < 0.001) and origin level (β = −0.0408 per ordinal step toward caudal origins, 95% CI − 0.0683 to − 0.0133, *p* = 0.005) were independent predictors, while sex was non-significant (*p* = 0.655). The full model accounted for 46% of the variance in left V1 TI (R² = 0.461; adjusted R² = 0.426). For the right V1 TI model, age (β = 0.0039, 95% CI 0.0014 to 0.0064, *p* = 0.003) and origin level (β = −0.0346, 95% CI − 0.0654 to − 0.0038, *p* = 0.029) were again independent predictors; sex was non-significant (*p* = 0.749). The right-side model R² was 0.272 (adjusted 0.225), reflecting both the smaller dynamic range of TI values on the right and the lower variability of origin levels on that side. Overall model fit was significant on both sides: left V1, F(3, 46) = 13.11, *p* < 0.001; right V1, F(3, 46) = 5.73, *p* = 0.002. No split-sample validation was performed; with 50 observations and three predictors, a hold-out partition would have been underpowered and unstable, and the models are therefore reported as descriptive of the present cohort rather than as validated prediction tools.

### V1 tortuosity and vertebral artery dominance

Among the 41 patients with a unilaterally dominant VA (excluding 9 codominant cases), V1 TI of the dominant vessel averaged 1.27 ± 0.22, compared with 1.24 ± 0.20 on the non-dominant side. The paired Wilcoxon test was non-significant (W = 381.0, *p* = 0.529). Likewise, the magnitude of bilateral TI asymmetry (|left − right TI|) showed no correlation with the magnitude of bilateral diameter asymmetry (Spearman ρ = 0.045, *p* = 0.759). No association between V1 tortuosity and calibre-based dominance was detected in this cohort; the 95% confidence interval for the paired difference (− 0.060 to + 0.118) does not exclude a difference of anatomically relevant magnitude. Left-sided predominance of tortuosity was present in 82% of patients, whereas calibre dominance was left-sided in 32%.

### Summary of findings

Four quantitative findings emerge from the morphometric analysis. (i) The left V1 is significantly more tortuous than the right (mean TI 1.37 vs. 1.20; *p* < 0.0001; d_z_ = 0.74; concordant in 82% of patients); both a longer centreline and a shorter straight-line on the left contribute additively to the bilateral asymmetry. (ii) V1 TI increases monotonically with age bilaterally (Spearman ρ = 0.44 right, 0.60 left), without any sex effect. (iii) V1 TI varies with VA origin level: more cranial origins are associated with substantially higher V1 TI (Spearman ρ = −0.64 left, − 0.37 right), an effect that retains independence from age in multivariable regression. (iv) No association was detected between V1 tortuosity and calibre-based VA dominance: there was no difference between dominant and non-dominant sides and no correlation between TI asymmetry and diameter asymmetry.

## Discussion

The left V1 was more tortuous than the right in 82% of patients (mean TI 1.37 versus 1.20; *p* < 0.0001; d_z = 0.74), and this asymmetry was explained by a more cranial VA origin set against a conserved bilateral C6 transverse-foraminal entry, not by calibre-based VA dominance. Age and origin level were independent predictors of tortuosity; sex was not.

### V1 tortuosity is not a manifestation of dominance

Left-sided V1 tortuosity has often been treated, implicitly, as a downstream consequence of left-sided VA dominance [14, 23]. The present data argue against that assumption. Within our cohort, the bilateral V1 tortuosity index showed no significant difference between the dominant and non-dominant sides (*p* = 0.53), and the magnitude of bilateral TI asymmetry was uncorrelated with the magnitude of bilateral diameter asymmetry (Spearman ρ = 0.045). Even in the largest meta-analysis to date, which pooled 32,153 vessels and reported left-sided dominance in roughly one in three patients [24], the directional alignment between dominance and tortuosity has never been quantitatively tested with bilateral, paired measurements; the present analysis is the first to do so directly. The result is unambiguous: V1 tortuosity and VA dominance are independent anatomical phenomena, and reasoning about one from the other is empirically unsupported.

### The origin–entry mismatch as a structural cause of laterality

**Fig. 7:**
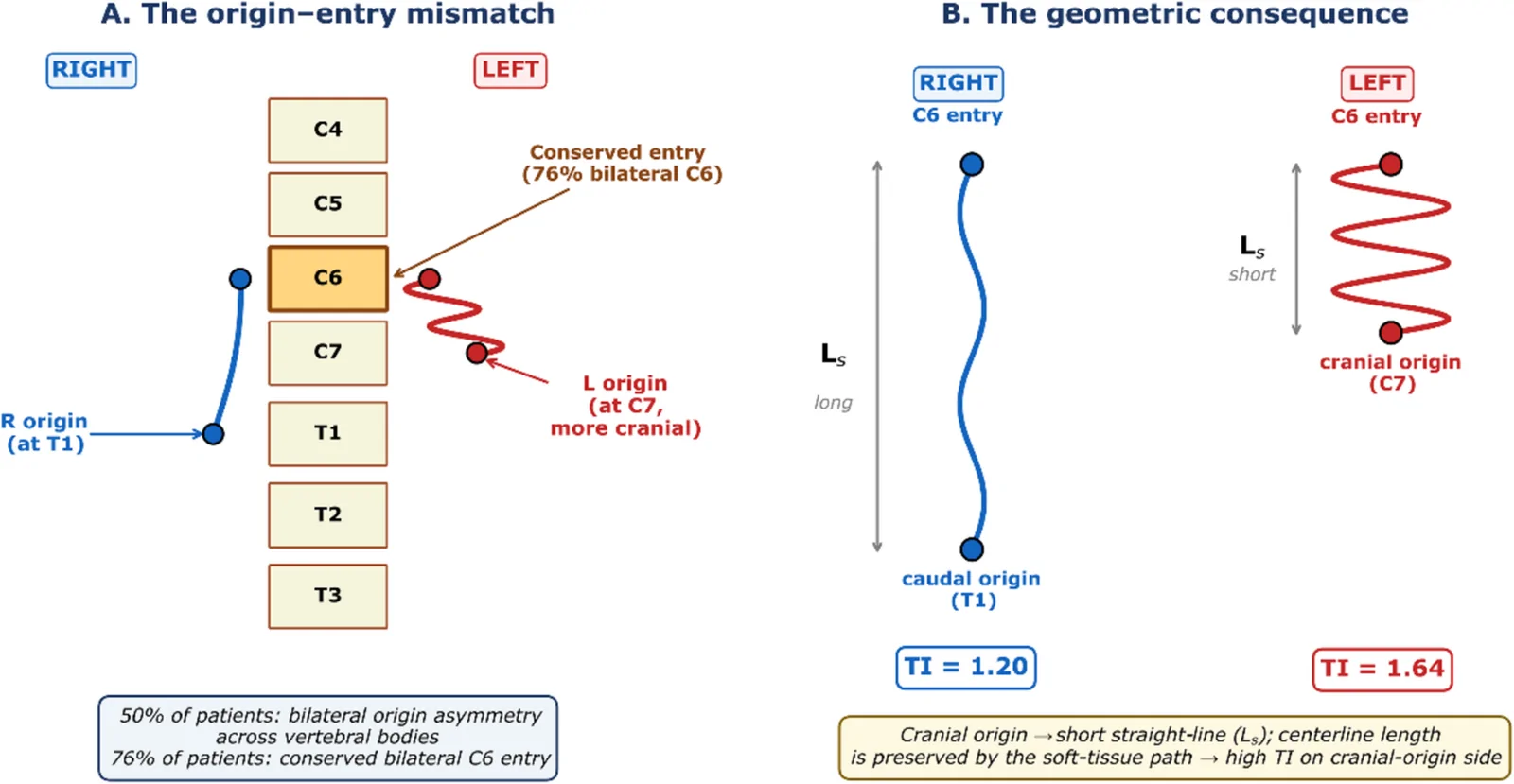
The origin–entry mismatch concept. (**A**) The vertebral artery enters the transverse foramen at a highly conserved C6 level bilaterally (76% bilateral C6 entry), while bilateral VA origin levels differ in 50% of patients; the left VA originates more cranially than the right in a subset of cases. (**B**) Geometric consequence: a cranial origin (e.g., left C7) produces a short straight-line distance (L_s_) between the origin and the conserved C6 entry, but the centreline (L_c_) is preserved by the artery’s soft-tissue path through the prevertebral region; the resulting tortuosity index (TI = L_c_/L_s_) is therefore high. A more caudal origin (e.g., right T1) produces a long straight-line distance, against which the centreline is comparable in length, and TI is low

The left V1 was more tortuous than the right in 82% of patients, on average by a Cohen’s d_z_ of 0.74, a large within-pair effect. The bilateral entry of the VA into the transverse foramen is highly conserved at C6 (76% bilateral C6 entry in our cohort, in agreement with the meta-analytic figure of 92% [24]). The bilateral origin of the VA from the subclavian artery, by contrast, is highly variable: 50% of our patients showed asymmetric bilateral origin levels across vertebral bodies, with the left VA originating more cranially in a subset of cases (Fig. 7A). Previous studies have documented variability in left VA origin from the aortic arch in 4.81% [24], 6% [7], and 13% [3] of cases, with corresponding variation in the level of transverse foraminal entry [3, 7, 30]. The present cohort extends this picture: even when both VAs originate conventionally from the subclavian artery, their bilateral origin levels are routinely discordant on a granular scale that previous studies have not measured.

The geometric consequence (Fig. 7B) is counterintuitive. A more cranial origin places the VA closer to its conserved foraminal entry, shortening the straight-line distance between the two endpoints. The centreline does not shorten proportionally, because the artery still has to negotiate the soft-tissue corridor between the longus colli muscle, the anterior scalene, and the prevertebral fascia, and because angulation through the costo-vertebral region adds path length. In our data, a left V1 originating at C7 had a mean straight-line length of 2.97 cm but a centreline of 4.88 cm, yielding TI = 1.64; a left V1 originating at T3 had a mean straight-line length of 8.41 cm and a centreline of 9.04 cm, yielding TI = 1.08. The cranial-origin segment is geometrically more tortuous, not because it is longer, but because its centreline is preserved while its chord is compressed. We name this configuration the origin–entry mismatch and propose it as the underlying anatomy for the left-sided predominance of V1 tortuosity documented here and in earlier qualitative literature [24, 28, 30].

### An embryological reading: why the origin is variable and the entry is not

The asymmetry between origin variability and entry conservation has a developmental rationale that is rarely articulated in the morphometric literature. In Padget’s classic reconstruction of the embryonic cranio-cervical arteries [19], extended in modern angiographic anatomy by Gregg and Gailloud [11], the VA arises from the cranial seven cervical intersegmental arteries through a process of selective involution: the dorsal cervical intersegmental branches anastomose longitudinally and the inter-segmental connections to the dorsal aorta regress, leaving the seventh cervical (or first thoracic) intersegmental artery as the definitive subclavian–VA junction. The longitudinal pre-vertebral anastomosis matures into the future V2–V3 course and, critically, reaches the cervical transverse foramina via a ventrolateral path that becomes the bony canal at C6. The site at which the artery enters this canal is therefore determined by the convergence of a developmental scaffold (the longitudinal anastomosis) and a skeletal scaffold (the foramen of C6), both of which are highly conserved across individuals. The site at which the artery leaves the systemic circulation, by contrast, depends on which of seven candidate intersegmental sources persists, and on the relative timing of involution of the others, including the primitive lateral basilovertebral anastomosis [11]. Variability is concentrated, by developmental design, at the proximal (origin) end of the segment.

### Acquired remodelling on a congenital substrate

The two strongest independent predictors of V1 TI in our multivariable models, age (β = 0.0081 per year on the left, *p* < 0.001; β = 0.0039 on the right, *p* = 0.003) and origin level (β = −0.041, *p* = 0.005, left; β = −0.035, *p* = 0.029, right), operate on different time scales. Origin level is fixed at the end of the embryonic period; age-related change in TI occurs over decades and reflects the cumulative impact of haemodynamic loading, arterial wall remodelling, atherosclerosis, and elastin–collagen disequilibrium documented for the carotid system [21, 25, 27] and now generalisable to the VA. Cervical arterial elongation has been associated with hypertension and BMI-driven intra-abdominal pressure changes [25, 27], with mild cognitive impairment [32] and with vertebrobasilar dolichoectasia, a progressive arterial disease whose prevalence rises with age and which carries an independent stroke risk [13]. In the present study, age was significant on both sides while sex was not, a pattern that aligns with a haemodynamic and remodelling-dominant mechanism rather than a hormonal one. The predictor set was restricted to age, sex and origin level because these were the only variables pre-specified as candidate determinants of V1 geometry and the only ones retrievable from an imaging-only retrospective dataset; with 50 observations, three predictors also represent the maximum consistent with conventional observations-per-predictor guidance. Vascular risk factors were not available (see Limitations).

The conceptual point is that these two drivers do not compete: they superimpose. A patient born with a cranial left VA origin starts adult life with a high baseline TI and accumulates additional tortuosity at the same ageing slope as everyone else. The implication is that a patient with apparently severe age-related left V1 tortuosity may, in fact, have had a structurally predisposed segment from birth, a distinction that has direct consequences for any clinical attempt to attribute tortuosity to atherosclerotic burden or to hypertension. Future studies that link tortuosity to clinical endpoints [23, 32] should covary on the origin level if they wish to isolate the acquired component.

### Limitations

Several limitations should be acknowledged. First, the right-side multivariable model accounted for only 27% of TI variance (left-side R² = 0.46), reflecting both the smaller dynamic range of TI on the right and the lower variability of right-side origins; unexplained variance on the right side remains substantial and may relate to factors not captured here, including aortic arch geometry, subclavian artery angulation, or local soft-tissue anatomy. Second, and most importantly for generalisability, the cohort was not population-based. All examinations had been requested for the diagnostic evaluation of suspected carotid arterial pathology, so the sample is older than the general population and enriched for cardiovascular risk; a further 12 of 62 screened examinations were excluded for insufficient V1 opacification, which may have selected against the most severely diseased vessels. The absolute TI values reported here should therefore not be treated as population reference values, and the age–TI slope may be steeper than in an unselected population. Vascular risk factors (hypertension, diabetes, smoking, body mass index, dyslipidaemia) were not retrievable for this imaging-only retrospective cohort, so the age coefficient should be read as a composite of chronological and vascular ageing. The origin-level effect is less exposed to this limitation, because VA origin level is determined during embryogenesis and cannot plausibly be modified by acquired vascular risk factors. Finally, the single-centre design and the sample size limit precision, particularly for origin levels represented by few patients.

### Future outlook

Because dominance and tortuosity are decoupled, haemodynamic mechanisms that operate through vessel calibre cannot be invoked to explain tortuosity-related risk. Tortuosity-driven phenomena, turbulent flow at angulations, intimal shear stress at coil apices, propensity to dissection [9, 16, 33], propensity to stent fracture [29], difficulty of endovascular catheterisation [5], and dolichoarteriopathy-related modulation of thrombolysis [1, 10], should therefore be assessed by direct geometric measurement of the vessel course rather than inferred from bilateral diameter ratios. We suggest that future work report V1 tortuosity as a separate anatomical variable in cervical CTA, with origin level recorded alongside calibre, and that studies linking tortuosity to clinical endpoints [23, 32] covary on origin level in order to isolate the acquired component of vascular elongation.

## Conclusion

Left-sided V1 tortuosity was associated with a more cranial subclavian origin set against a conserved C6 transverse foraminal entry, a configuration we term the origin–entry mismatch, and no association with calibre-defined dominance was detected. V1 tortuosity should therefore be reported as a separate anatomical variable in cervical CTA, with origin level recorded alongside calibre.

## Supplementary Information

Below is the link to the electronic supplementary material.

Supplementary Material 1

## Data Availability

The datasets used and analyzed during the current study are available from the corresponding author upon reasonable request.
