# Supplementary material for: Origin–entry geometry and left-sided V1 vertebral artery tortuosity: a retrospective cross-sectional CT angiography study
Source: Neuroradiology. 2026 Sep 3;68(9):2587–600. doi: 10.1007/s00234-026-04180-9 (PMC13680300; doi:10.1007/s00234-026-04180-9)
Supplement: Supplementary file 1 [file 234_2026_4180_MOESM1_ESM.docx]

**Online Resource 1**

**Origin–entry geometry and left-sided V1 vertebral artery tortuosity: a retrospective cross-sectional CT angiography study**

**Supplementary Methods**

**S1. Image post-processing environment and display settings**

All post-processing and measurements were performed in Horos (version 4.0; The Horos Project, https://horosproject.org), an open-source DICOM viewer derived from OsiriX. Horos was selected because (i) it is publicly available and therefore makes the image-processing pipeline of the study fully reproducible by other investigators, (ii) it provides full multi-planar reformation, maximum-intensity projection, three-dimensional volume rendering and curved multi-planar reformation functionality, and (iii) its measurement tools have been validated against industry-standard vendor workstations in prior peer-reviewed cardiovascular imaging work [12]. All measurements were performed on a calibrated 27-inch diagnostic monitor in a darkened reading-room environment. Window width and window level were adjusted manually for optimal vessel-versus-background contrast at each measurement step (typical values: WW 600–800 HU, WL 150–200 HU).

**S2. Manual centreline tracing of the V1 vertebral artery segment**

**Step 1. Identification of segment endpoints.** The proximal endpoint was placed at the centre of the vertebral artery ostium on the axial slice in which the artery first appeared as a discrete contrast-enhanced lumen separate from the subclavian artery. The distal endpoint was placed at the centre of the vertebral artery lumen on the axial slice in which the vessel first entered the transverse foramen of its respective cervical vertebra (most commonly C6; see section 2.4.6 of the main text). Both endpoints were confirmed against the corresponding sagittal and coronal cross-sections; in cases of ambiguity, the more proximal candidate slice was retained for the proximal endpoint and the more distal candidate slice for the distal endpoint, ensuring that the full V1 length was captured.

**Step 2. Control-point placement.** Between the two endpoints, additional control points were placed manually at approximately 3–5 mm intervals along the visualised vessel lumen. Each control point was positioned at the geometric centre of the contrast-enhanced lumen on the axial cross-section perpendicular to the local vessel axis at that level. In regions of pronounced curvature or angulation, control-point spacing was reduced to approximately 2 mm to capture the geometric detail of the bend without introducing chord-induced error. In total, between 12 and 18 control points were placed per V1 segment, depending on segment length and tortuosity.

**Step 3. Curved-path reconstruction and quality control.** The V1 course was reconstructed using the Horos curved-MPR/path tool by placing sequential centre points along the contrast-enhanced lumen from the vertebral artery origin to the transverse-foramen entry point, in accordance with the control-point protocol described in Step 2. The path was inspected and adjusted iteratively in axial, coronal and sagittal reformations to remain centred within the vessel lumen. Additional points were placed in regions of angulation or pronounced curvature to reduce chord-length approximation error. The reconstructed path was used as the operational centreline for length measurement and visual export.

**Step 4. Length computation.** The V1 centreline length, denoted Lc, was obtained as the curvilinear length of the manually defined path between the proximal and distal endpoints, derived from the Horos curved path. The straight-line length, denoted Ls, was measured as the direct distance between endpoints on a standardised oblique multiplanar reconstruction passing through the vertebral artery origin and the transverse foramen entry point. The tortuosity index was calculated as TI = Lc/Ls. Both length measurements were recorded in centimetres.

**Table S1**

**Table S1.** CT angiography acquisition and image reconstruction parameters. Referenced from section 2.3 of the main text.

| **Parameter** | **Value** |
| --- | --- |
| Scanner | Siemens SOMATOM Perspective, 32-slice (Siemens Healthineers, Forchheim, Germany) |
| Tube voltage | 120 kVp |
| Tube current | Automatic tube current modulation (CARE Dose4D); reference 150 mAs |
| Detector collimation | 32 × 0.6 mm |
| Rotation time | 0.6 s |
| Pitch | 0.85 |
| Reconstructed slice thickness | 0.75 mm with 50% overlap |
| Reconstruction kernel | B30f (medium-smooth), routine for soft-tissue and vascular display |
| Field of view | Aortic arch to the circle of Willis |
| Contrast medium | Non-ionic iodinated contrast (Iohexol 350 mg I/mL or equivalent), 80–100 mL, injected at 4–5 mL/s through an antecubital (brachial) intravenous line, followed by a 30 mL saline chaser at the same rate |
| Bolus tracking | Region-of-interest in the ascending aorta; trigger threshold 100 HU; acquisition delay 4 s |
| Post-processing reconstructions | Multi-planar reformations (MPR), maximum-intensity projections (MIP), and three-dimensional volume rendering (3D-VR) |

**Acquisition procedure.** Following standard institutional practice, scout images were acquired in antero-posterior and lateral projections to plan the diagnostic acquisition. The arterial-phase scan was acquired in the cranio-caudal direction (vertex to aortic arch) and triggered by automatic bolus tracking with a region of interest placed in the ascending aorta (threshold 100 HU; minimum 4-second post-trigger delay). Source data were reconstructed at a 0.75-mm slice thickness with 50% inter-slice overlap, in accordance with current best practice for vascular morphometry in cervical CTA, to produce isotropic or near-isotropic voxels suitable for downstream multi-planar reformation, maximum-intensity projection (MIP) and three-dimensional volume rendering (3D-VR). All reconstructions were performed at the scanner console and exported as DICOM datasets.

kVp = peak kilovoltage; mAs = milliampere-seconds; HU = Hounsfield units; MPR = multi-planar reformation; MIP = maximum-intensity projection; 3D-VR = three-dimensional volume rendering.
